# Urinary Excretion of *N*^1^-Methylnicotinamide, as a Biomarker of Niacin Status, and Mortality in Renal Transplant Recipients

**DOI:** 10.3390/jcm8111948

**Published:** 2019-11-12

**Authors:** Carolien P.J. Deen, Anna van der Veen, Martijn van Faassen, Isidor Minović, António W. Gomes-Neto, Johanna M. Geleijnse, Karin J. Borgonjen-van den Berg, Ido P. Kema, Stephan J.L. Bakker

**Affiliations:** 1 Department of Internal Medicine, University of Groningen, University Medical Center Groningen, 9713 GZ Groningen, The Netherlands; a.w.gomes.neto@umcg.nl (A.W.G.-N.);; 2 Department of Laboratory Medicine, University of Groningen, University Medical Center Groningen, 9713 GZ Groningen, The Netherlands; a.van.der.veen03@umcg.nl (A.v.d.V.); h.j.r.van.faassen@umcg.nl (M.v.F.); i.minovic@umcg.nl (I.M.); i.p.kema@umcg.nl (I.P.K.); 3 Top Institute Food and Nutrition, 6709 PA Wageningen, The Netherlands; 4 TransplantLines Food and Nutrition Biobank and Cohort Study, University of Groningen, University Medical Center Groningen, 9713 GZ Groningen, The Netherlands; 5 Division of Human Nutrition and Health, Wageningen University, 6708 PB Wageningen, The Netherlands; marianne.geleijnse@wur.nl (J.M.G.); karin.borgonjen@wur.nl (K.J.B.-v.d.B.)

**Keywords:** urinary excretion of *N*^1^-methylnicotinamide, kidney transplantation, mortality, niacin status, dietary intake, tryptophan, vitamin B_3_

## Abstract

Renal transplant recipients (RTR) commonly suffer from vitamin B_6_ deficiency and its functional consequences add to an association with poor long-term outcome. It is unknown whether niacin status is affected in RTR and, if so, whether this affects clinical outcomes, as vitamin B_6_ is a cofactor in nicotinamide biosynthesis. We compared 24-h urinary excretion of *N*^1^-methylnicotinamide (*N*^1^-MN) as a biomarker of niacin status in RTR with that in healthy controls, in relation to dietary intake of tryptophan and niacin as well as vitamin B_6_ status, and investigated whether niacin status is associated with the risk of premature all-cause mortality in RTR. In a prospective cohort of 660 stable RTR with a median follow-up of 5.4 (4.7–6.1) years and 275 healthy kidney donors, 24-h urinary excretion of *N*^1^-MN was measured with liquid chromatography-tandem mass spectrometry LC-MS/MS. Dietary intake was assessed by food frequency questionnaires. Prospective associations of *N*^1^-MN excretion with mortality were investigated by Cox regression analyses. Median *N*^1^-MN excretion was 22.0 (15.8–31.8) μmol/day in RTR, compared to 41.1 (31.6–57.2) μmol/day in healthy kidney donors (*p* < 0.001). This difference was independent of dietary intake of tryptophan (1059 ± 271 and 1089 ± 308 mg/day; *p* = 0.19), niacin (17.9 ± 5.2 and 19.2 ± 6.2 mg/day; *p* < 0.001), plasma vitamin B_6_ (29.0 (17.5–49.5), and 42.0 (29.8–60.3) nmol/L; *p* < 0.001), respectively. *N*^1^-MN excretion was inversely associated with the risk of all-cause mortality in RTR (HR 0.57; 95% CI 0.45–0.71; *p* < 0.001), independent of potential confounders. RTR excrete less *N*^1^-MN in 24-h urine than healthy controls, and our data suggest that this difference cannot be attributed to lower dietary intake of tryptophan and niacin, nor vitamin B_6_ status. Importantly, lower 24-h urinary excretion of *N*^1^-MN is independently associated with a higher risk of premature all-cause mortality in RTR.

## 1. Introduction

Kidney transplantation is the preferred treatment for end-stage renal disease in terms of survival, quality of life and costs [1,2]. Advances in transplantation medicine have lifted the 1-year patient survival higher than 90% [3]. While short-term patient outcomes are continuing to improve, the long-term posttransplant survival has remained largely unchanged over the past few decades [4]. Compared with the general population, renal transplant recipients (RTR) are at highly increased risk of premature mortality [5]. Improving perspectives relies on the management of modifiable factors that impact long-term outcome in RTR, of which nutrition is increasingly acknowledged [6,7].

Recently, we found that RTR commonly suffer from vitamin B_6_ deficiency and its functional consequences that add to an association with poor long-term outcomes [8]. As vitamin B_6_ is an essential cofactor of key enzymes involved in de novo biosynthesis of nicotinamide from tryptophan [9], niacin deficiency might be lurking in these patients as well. Nicotinamide, nicotinic acid, and nicotinamide riboside are collectively referred to as niacin or vitamin B_3_, and are precursors of the metabolically active NAD^+^. Besides dietary intake of pre-formed niacin, the so-called tryptophan-nicotinamide pathway is critical to maintaining niacin status [10]. Ongoing NAD^+^ supply from its metabolic precursors, collectively referred to as “niacin equivalents”, is required to provide reducing equivalents for energy metabolism and substrates of NAD^+^ consuming enzymes [11]. NAD^+^ is catabolized to *N*^1^-methylnicotinamide (*N*^1^-MN) through methylation of nicotinamide in the liver, and the 24-h urinary excretion of *N*^1^-MN is considered the most reliable biomarker of niacin status [12,13,14].

It is unknown whether niacin status is affected in RTR and, if so, whether this affects clinical outcomes. Hence, this study aims to compare 24-h urinary excretion of *N*^1^-MN in RTR with that in healthy kidney donors, in relation to dietary intake of tryptophan and niacin as well as vitamin B_6_ status, and to investigate whether niacin status is associated with the risk of premature all-cause mortality in RTR.

## 2. Materials and Methods

### 2.1. Study Population

This prospective study was conducted in a well-characterized, single-center cohort of 707 RTR (aged ≥18 years) with a functioning graft for at least 1 year who visited the outpatient clinic of the University Medical Center Groningen, Groningen, the Netherlands, between 2008 and 2011 [15,16,17]. As a control group, 367 healthy kidney donors were included who participated in a screening program before kidney donation. Signed informed consent was obtained from all participating subjects and the study protocol was approved by the institutional review board (METc 2008/186) adhering to the Declaration of Helsinki. Exclusion of subjects with missing biomaterial or niacin supplementation use left 660 RTR and 275 kidney donors eligible for statistical analyses (Figure S1).

### 2.2. Data Collection

All baseline measurements were obtained during a morning visit to the outpatient clinic. Participants were instructed to collect a 24-h urine sample on the day before their visit, and to fast overnight for 8 to 12 h. Urine samples were collected under oil, and chlorhexidine was added as an antiseptic agent. Fasting blood samples were drawn after completion of the urine collection. Blood was collected in a series of evacuated tubes with different additives (Vacutainer^®^; BD, Franklin Lakes, NJ, USA) for preparation of plasma and serum. Body composition and hemodynamic parameters were measured according to a previously described, strict protocol [15]. Serum parameters, including lipid, inflammation, and glucose homeostasis variables were measured with spectrophotometric-based routine clinical laboratory methods (Roche Diagnostics, Rotkreuz, Switzerland). Diabetes was diagnosed if fasting plasma glucose was ≥7.0 mmol/L or antidiabetic medication was used [15]. Plasma vitamin B_6_ was determined as its principal, metabolically active form pyridoxal-5′-phosphate using a HPLC method (Waters Alliance, Milford, MA, USA) with fluorescence detection (JASCO, Inc., Easton, MD, USA) [8].

Renal function was assessed by estimation of the glomerular filtration rate (eGFR) and detection of proteinuria. The eGFR was calculated using the combined creatinine and cystatin C-based Chronic Kidney Disease Epidemiology Collaboration equation [18], which has been shown to be the most accurate equation in RTR [19]. Proteinuria was diagnosed if total urinary protein excretion was ≥0.5 g/day as measured by a biuret reaction-based assay (MEGA AU510; Merck Diagnostica, Darmstadt, Germany).

Dietary intake including tryptophan and niacin intakes was assessed with a validated semi-quantitative food frequency questionnaire (FFQ) [20,21,22]. The self-administered questionnaire was filled out at home and inquired about 177 food items during the last month, taking seasonal variations into account. During the visit to the outpatient clinic, the FFQ was checked for completeness by a trained researcher and inconsistent answers were verified with the participant. The FFQ was validated for RTR as previously reported [16]. Dietary data were converted into daily nutrient intake using the Dutch Food Composition Table of 2006 [23]. Alcohol consumption and smoking behavior were assessed with a separate questionnaire [6]. Additional data on medical history and use of medication and vitamin supplements were obtained from medical records [6].

### 2.3. Assessment of N^1^-MN Excretion

Measurement of *N*^1^-MN concentration was performed with a validated liquid chromatography (Luna HILIC column; Phenomenex, Torrance, CA, USA) isotope dilution-tandem mass spectrometry (LC-MS/MS) (Quattro Premier; Waters, Milford, MA, USA) method, as described previously [24]. The 24-h urinary excretion of *N*^1^-MN (μmol/day) was obtained after multiplying *N*^1^-MN concentration (μmol/L) by total urine volume calculated from weight (L/day). The reference range of *N*^1^-MN excretion in healthy individuals was previously established at 17.3–115 μmol/day [24].

### 2.4. Clinical Endpoints

The primary outcome of this study was all-cause mortality which was recorded until 30 September 2015 with no loss due to follow-up. RTR status was kept up-to-date through the continuous surveillance system of the outpatient program.

### 2.5. Statistical Analysis

Data are presented as the mean ± SD, median (IQR) and absolute number (percentage) for normally distributed, skewed, and nominal data, respectively. Assumptions for normality were checked by visual judgments of the corresponding frequency distribution and Q-Q plot.

Baseline characteristics of RTR and healthy kidney donors were compared by means of *t*, Mann-Whitney, and Chi-Square tests. Niacin status in RTR and healthy kidney donors was compared by linear regression analyses of 2-base log-transformed *N*^1^-MN excretion, with subsequent cumulative adjustment for age and sex (model 1), eGFR (model 2) and intake of energy, tryptophan, and niacin and plasma vitamin B_6_ (model 3).

RTR characteristics were divided into tertiles of *N*^1^-MN excretion stratified by sex (T1, T2, and T3) and compared by means of ANOVA, Kruskal-Wallis, and Chi-Square tests.

For prospective analyses, a Cox proportional hazards regression model for all-cause mortality outcome was fitted to *N*^1^-MN excretion as a sex-stratified tertile-based categorical variable, as well as a continuous variable adjusted for sex (model 1). Confounding was controlled for by including potential confounders as covariates in the regression model. Crude associations were adjusted cumulatively for age (model 2), smoking and body surface area (model 3) and, to prevent overfitting, additionally for intake of alcohol and energy and plasma vitamin B_6_ (model 4), kidney function (model 5), medication use (model 6), and high-sensitivity C-reactive protein (hs-CRP) (model 7). Variables that could lie in the causal pathway of *N*^1^-MN excretion and all-cause mortality were not adjusted for because this might obscure otherwise existing associations unintentionally. Assumptions of proportionality of the hazard functions and the linearity of log-hazards were checked by visual judgements of Kaplan Meier plots of the survival and log-survival function entering the sex-stratified *N*^1^-MN excretion tertile group variable.

In secondary analyses, effect modification was assessed by including the cross product term of each potential confounder and 2-base log-transformed *N*^1^-MN excretion in the Cox regression model adjusted for age and sex (model 2). Subsequent stratified analyses were performed for subgroups of significant effect modifiers on the association of *N*^1^-MN excretion with all-cause mortality.

For all statistical analyses, a two-sided *p*-value of less than 0.05 was considered to indicate statistical significance and SPSS Statistics version 23.0 (IBM, Armonk, NY, USA) was used as software.

## 3. Results

### 3.1. Baseline Characteristics and Comparison of N^1^-MN Excretion

**Figure 1:**
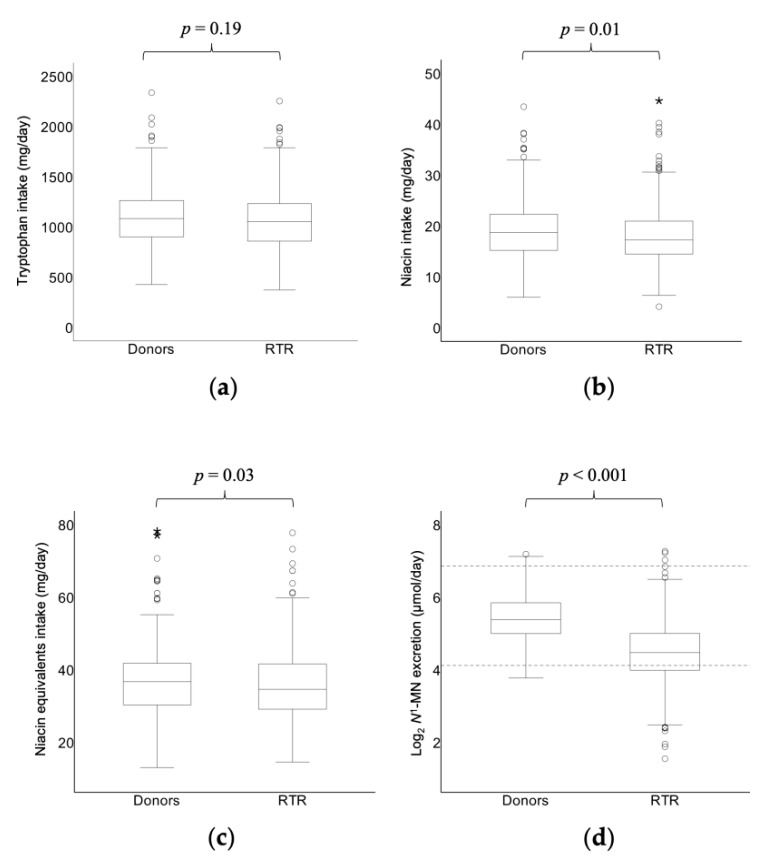
Box plots of dietary intake of (**a**) tryptophan, (**b**) niacin and (**c**) niacin equivalents and (**d**) log_2_ 24-h urinary excretion of *N*^1^-MN in RTR compared to that in healthy kidney donors. Boxes, bars and whiskers represent IQRs, medians and values <1.5 × IQR, respectively, whereas outliers (1.5–3 × IQR) are indicated by circles and extreme outliers (>3 × IQR) by asterisks. Log_2_ of the lower and upper bound of the reference range of *N*^1^-MN excretion in healthy individuals (17.3–115.0) μmol/day [24] are indicated with dotted lines (**d**). *p*-value for difference between RTR and donors was tested by t and Mann-Whitney tests for normally and skewed distributed continuous variables, respectively. Intake of niacin equivalents was calculated by adding up niacin and one-sixtieth of tryptophan intake. *N*^1^-MN, *N*^1^-methylnicotinamide; RTR, renal transplant recipients.

This study included 660 stable RTR (57% male; mean age 53.0 ± 12.7 years), at a median time of 5.6 (2.0–12.0) years after transplantation, and 275 healthy kidney donors (41% male; mean age 53.3 ± 10.7 years) (Table 1). Intake of tryptophan was similar in both groups (1059 ± 271 and 1089 ± 308 mg/day, respectively; *p* = 0.19), while intake of niacin was lower in RTR than in kidney donors (17.9 ± 5.2 and 19.2 ± 6.2 mg/day, respectively; *p* = 0.01). Taken together, intake of niacin equivalents was lower in RTR than in kidney donors (35.6 ± 9.2 mg/day and 37.4 ± 10.8, respectively; *p* = 0.03) (Figure 1). All RTR and kidney donors complied with the recommended daily intake that is set at a minimum of 6.6 niacin equivalents per 1000 kcal (≥ 9.6 and ≥ 11.7 mg/1000 kcal, respectively) [12]. As previously reported, RTR had significantly lower plasma vitamin B_6_ compared to kidney donors (29.0 (17.5–49.5) and 42.0 (29.8–60.3) nmol/L, respectively; *p* < 0.001). Median *N*^1^-MN excretion was 22.0 (15.8–31.8) μmol/day in RTR, compared to 41.1 (31.6–57.2) μmol/day in kidney donors (*p* < 0.001) (Figure 1). Furthermore, urinary excretion of *N*^1^-MN was below the reference limit of 17.3 μmol/day in 202 (31%) RTR, against 4 (2%) kidney donors. The difference in *N*^1^-MN excretion between RTR and kidney donors was independent of age, sex, eGFR, intake of energy, tryptophan, and niacin and plasma vitamin B_6_ (Table 2). Cyclosporine, azathioprine, and anticonvulsants were used by, respectively, 253 (38%), 112 (17%) of 19 (3%) of RTR, and none of the controls received drugs that are known to potentially affect niacin status.

RTR characteristics across tertiles of sex-stratified *N*^1^-MN excretion (M: <19.2, 19.2–28.8, >28.8 μmol/day; F: <16.1, 16.1–25.6, >25.6 μmol/day in T1, T2, and T3, respectively) are shown in Table 3. Age and the presence of acetylsalicylic acid, proton pump inhibitors, diuretics and post mortem donors were lower with increasing tertiles of *N*^1^-MN excretion, while intake of alcohol, energy, tryptophan and niacin, plasma vitamin B_6_, kidney function and the presence of proliferation inhibitors and primary glomerular disease were higher with increasing tertiles of *N*^1^-MN excretion.

### 3.2. N^1^-MN Excretion and Mortality

**Figure 2:**
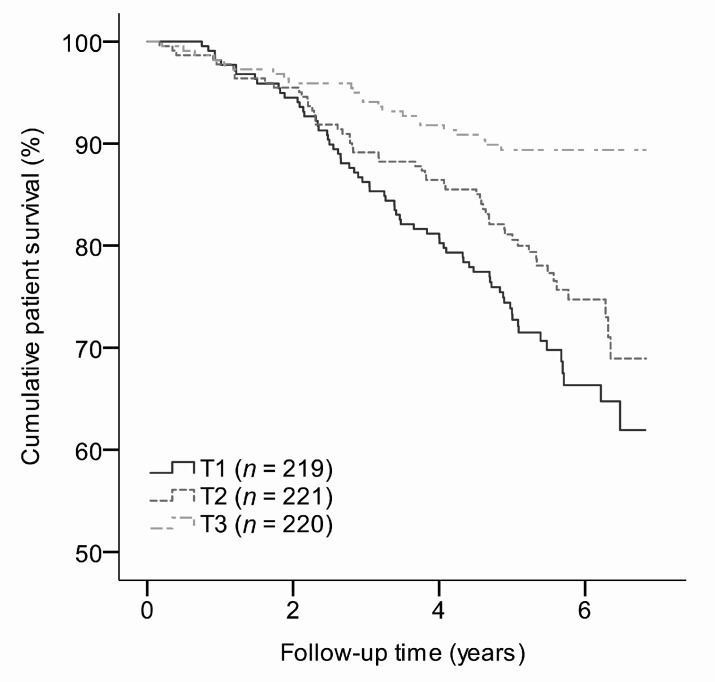
Survival curves for all-cause mortality in RTR according to tertiles of sex-stratified *N*^1^-MN excretion. *N*^1^-MN excretion was <19.2, 19.2–28.8, and >28.8 μmol/day for males, and <16.1, 16.1–25.6 and >25.6 μmol/day for females in T1, T2, and T3, respectively. *N*^1^-MN, *N*^1^-methylnicotinamide; RTR, renal transplant recipients.

During a median follow-up time of 5.4 (4.7–6.1) years, 143 (22%) RTR died. The risk of all-cause mortality increased with lower tertiles of *N*^1^-MN excretion, as depicted by Kaplan-Meier curves (Figure 2). Cox regression analyses revealed an inverse association of *N*^1^-MN excretion with all-cause mortality (Model 2: HR 0.57; 95% CI 0.45–0.71; *p* < 0.001), independent of potential confounders (Table 4). The same held for analyses across tertiles of sex-stratified *N*^1^-MN excretion (Table 4). RTR in the lowest and middle tertiles were at higher risk of all-cause mortality compared to those in the highest tertile as reference (Model 2: HR 2.68; 95% CI 1.67–4.33; *p* < 0.001 and HR 2.04; 95% CI 1.25–3.34; *p* = 0.004, respectively), independent of potential confounders (Table 4).

Secondary analyses exposed significant effect modification of hs-CRP on the association of *N*^1^-MN excretion with all-cause mortality (*p* = 0.05), independent of age and sex. The inverse association of *N*^1^-MN excretion with all-cause mortality was stronger for individuals in the subgroup with serum hs-CRP <2.4 mg/L (HR 0.47; 95% CI 0.35–0.64; *p* < 0.001), than in the subgroup with serum hs-CRP ≥2.4 mg/L (HR 0.70; 95% CI 0.50–0.96; *p* = 0.03) according to subsequent stratified analysis.

## 4. Discussion

In this large prospective cohort study, we showed that RTR excrete less *N*^1^-MN in 24-h urine than healthy controls and our data suggest that this difference cannot be attributed to lower dietary intake of tryptophan and niacin, nor vitamin B_6_ status. Furthermore, lower 24-h urinary excretion of *N*^1^-MN as a biomarker of niacin status was independently associated with a higher risk of premature all-cause mortality in RTR.

To the best of our knowledge, niacin status has not been studied within the context of kidney transplantation and its concomitant long-term implications yet. In fact, prospective data on the urinary excretion of *N*^1^-MN have been limited to one previous study in patients recovering from leukemia treatment [25]. Studies on niacin nutrition in relation to prospective outcomes are likewise scarce, as the prevailing intake of niacin equivalents is suggested to be not sufficiently low to compromise survival. Presumed health benefits of niacin are pharmacological rather than physiological [26,27,28,29], although higher survival with higher niacin intake in elderly has been reported previously [30] in congruence with our findings.

Niacin is considered the least critical vitamin to meet the recommendations through dietary intake in western societies [31], as niacin equivalents are found in a wide range of foods [12]. In line with this, dietary intake of niacin equivalents was sufficient according to WHO guidelines in all RTR and healthy kidney donors, while we found that urinary excretion of *N*^1^-MN was commonly below the established reference bound in RTR. The observed disparity of *N*^1^-MN excretion between RTR and healthy kidney donors could moreover not be explained by lower dietary intake of niacin equivalents in RTR in the present study.

The fact that we found a positive association of plasma vitamin B_6_ concentration with *N*^1^-MN excretion strengthens our hypothesis that inadequacies of this cofactor might affect niacin status in RTR. Adjustment for plasma vitamin B_6_, however, neither did alter the discrepancy of *N*^1^-MN excretion between RTR and healthy kidney donors. Therefore, one should consider other factors that could interfere with *N*^1^-MN excretion as a biomarker of niacin status, and add to poor long-term outcome in RTR.

Whereas secondary dietary inadequacies may interrupt niacin metabolism, this also holds for certain medications including specific antituberculosis, anticonvulsant and antiproliferative drugs, as well as cyclosporine and azathioprine [32,33,34], which are common immunosuppressant drugs in RTR, although in our population those did not appear to affect *N*^1^-MN excretion.

We can furthermore speculate on the presence of enhanced consumption of tryptophan for protein biosynthesis at the cost of niacin status in RTR. Interestingly, tryptophan is argued to be quantitatively the most important NAD^+^ precursor, as it is more effective in elevating liver NAD^+^ and urinary excretion of *N*^1^-MN than the salvageable precursors [35,36,37,38]. The tryptophan-nicotinamide pathway is, however, mainly regulated by tryptophan intake rather than niacin status, since the generally accepted conversion ratio of 60:1 falls when dietary tryptophan is limiting [39]. Indeed, tryptophan is used primarily for protein biosynthesis and only after nitrogen balance has been achieved for the nicotinamide pathway [40]. This allows us to speculate on protein catabolism and negative protein balance as part of protein-energy wasting in RTR, engendered by metabolic derangement, systemic inflammation, acidemia, and the use of immunosuppressive drugs, to induce tryptophan consumption for protein synthesis in this population [41,42]. However, as our study was not designed to assess protein-energy wasting, we cannot conclusively address such an effect on *N*^1^-MN excretion in RTR.

On the contrary, the tryptophan-nicotinamide pathway is implicated in disease states in which systemic inflammation is present, by the enhanced action of indoleamine 2,3-dioxygenase in response to inflammatory cytokines and mediators. This upregulation of tryptophan degradation towards nicotinamide is known to yield relativity large amounts of quinolinic acid to fuel NAD^+^-consuming poly (ADP-ribose) polymerase (PARP) reaction in response to immune-related (oxidative) damage [35]. Although we observed lower serum hs-CRP levels as a low-grade inflammation biomarker with higher tertiles of *N*^1^-MN excretion, this difference did not reach significance.

Finally, the renal clearance of *N*^1^-MN itself can also be affected by several factors and not in the least by impaired kidney function. In fact, *N*^1^-MN is eliminated almost exclusively by the kidneys, being partly excreted partly by glomerular filtration and partly by tubular secretion with negligible and saturable tubular reabsorption [43]. Whereas renal clearance of *N*^1^-MN has been investigated as a model of renal secretory function [43] and to predict renal clearance of cationic drugs in renal insufficiency [44], plasma concentrations are suggested to be less sensitive to kidney function because of the contribution of aldehyde oxidase to *N*^1^-MN clearance, yielding *N*^1^-methyl-2-pyridone-5-carboxamide (2Py) [45]. Although our findings appeared independent of kidney function, future studies are warranted to rule out enhanced oxidative metabolism, causing a shift towards urinary excretion of 2Py in this population.

Regarding potential mechanisms for the association of *N*^1^-MN excretion with mortality, NAD^+^ homeostasis has been linked to increased resistance against a range of pathophysiological processes that are predominant and impact poor long-term outcome in RTR, including cardiovascular, inflammatory, malignant and metabolic disorders [46]. The availability of NAD^+^ is determined by its production from niacin equivalents, as well as its degradation in NAD^+^ consuming activities [47]. NAD^+^ levels remain constant when used as a coenzyme, being recycled back and forth between its oxidized and reduced forms [11], but are depleted by three distinct classes of enzymes that consume NAD^+^ as a substrate: PARP, cyclic ADP ribose synthases (CD38 and CD157), and sirtuins [48]. Excessive activation of PARP and CD38 is induced by stresses such as inflammation, oxidative stress and DNA damage that are predominant in in RTR [48,49]. As a result, NAD^+^ availability might become limiting for beneficial sirtuin activities; in particular with lower niacin status. These beneficial effects of sirtuins have been described more specifically for renal diseases, including renoprotective effects by inhibition of renal cell apoptosis, inflammation, and fibrosis and regulation of mitochondrial function and glucose, lipid, and energy metabolism [50,51,52,53].

Whereas we did not find an association of *N*^1^-MN excretion with hs-CRP, this low-grade inflammation biomarker appeared to affect the magnitude of the inverse association of *N*^1^-MN excretion with all-cause mortality. Although we can only speculate on the underlying mechanism, earlier mentioned inflammation-related overconsumption of NAD^+^ limiting its downstream beneficial activities might at least partly explain the lower protective effect of niacin status on mortality in the subgroup with higher serum hs-CRP levels.

The current study should be interpreted within its strengths and limitations. First, its observational nature prohibits causal inferences, but it also did not allow us to draw conclusions on underlying mechanisms of lower *N*^1^-MN excretion in RTR and its contribution to worse survival. Second, the generalizability of our findings might be compromised by overrepresentation of Caucasian individuals from a single center, despite being controlled for by the inclusion of a large, representative control group. Third, the reliability of FFQ data is subject to sources of measurement error, including recall and social desirability biases and limitations in food composition databases [54]. Higher similarity in dietary sources could be achieved by including spouses as a control group. Finally, the present study is confined to the 24-h urinary excretion of *N*^1^-MN as the recommended biomarker of niacin nutritional status by authorities, including the WHO and the European Food Safety Authority [12,13,14]. Future studies are, however, encouraged to elaborate on plasma concentrations of niacin and its metabolites, or NAD^+^ and the ratio of NAD^+^ to NADP^+^ in erythrocytes as additional indices of niacin status. Although observational evidence is inherent to limitations, prospective cohort studies provide a strong design to address nutritional status and health outcome associations over a long period of time. Strengths of our study include its large sample size, with a sufficient number of incident cases and no loss to follow-up, and therefore minimizing the risk of bias in the assessment of outcome. The extensive characterization of participants, moreover, allowed us to control for confounding and effect modification in estimates of the effect.

## 5. Conclusions

In conclusion, 24-h urinary excretion of *N*^1^-MN as a biomarker of niacin status is lower in RTR than in healthy controls, and other factors than dietary intake of niacin equivalents and vitamin B_6_ status appear to reinforce this discrepancy. Importantly, 24-h urinary excretion of *N*^1^-MN is inversely associated with a higher risk of premature all-cause mortality in RTR and niacin status is therefore revealed as a potential target for nutritional strategies to improve long-term outcome after kidney transplantation. However, further research is warranted to unravel underlying mechanisms that potentially interfere with *N*^1^-MN excretion in RTR, and to strengthen causal inferences for health outcomes to support dietary recommendation.

## Figures and Tables

**Table 1:** Baseline characteristics of stable RTR compared to that in healthy kidney donors ^1^.

Variable	Donors *n* = 275	RTR *n* = 660	*p*-Value ^2^
Age, years	53.3 ± 10.7	53.0 ± 12.7	0.68
Male, *n* (%)	112 (41)	379 (57)	0.001
Body surface area, m^2^	1.9 ± 0.2	1.9 ± 0.2	0.90
Current smoker, *n* (%)	39 (14)	78 (12)	<0.001
Alcohol intake, g/day	6.7 (1.1–16.4)	3.1 (0.1–11.9)	<0.001
Energy intake, kcal/day	2295 ± 746	2182 ± 642	0.04
Niacin equivalents intake, mg/day ^3^	37.4 ± 10.8	35.6 ± 9.2	0.03
Tryptophan intake, mg/day	1089 ± 308	1059 ± 271	0.19
Niacin intake, mg/day	19.2 ± 6.2	17.9 ± 5.2	0.01
*N*^1^-MN excretion, μmol/day	41.4 (31.6–57.2)	22.0 (15.8–31.8)	<0.001
<17.3 μmol/day, *n* (%)	4 (2)	202 (31)	0.03
Plasma vitamin B_6_ (nmol/L)	42.0 (29.8–60.3)	29.0 (17.5–49.5)	<0.001
Systolic blood pressure, mmHg	125.1 ± 13.9	135.8 ± 17.3	<0.001
Diastolic blood pressure, mmHg	75.6 ± 9.1	82.5 ± 11.0	<0.001
Triglycerides, mmol/L	1.2 (0.9–1.7)	1.7 (1.2–2.3)	<0.001
HbA1c, (%)	5.6 (5.4–5.8)	5.8 (5.5–6.2)	<0.001
eGFR, ml/min/1.73 m^2^	91.0 ± 14.2	53.0 ± 20.0	<0.001
Acetylsalicylic acid, *n* (%)	4 (2)	127 (19)	<0.001
Proton pump inhibitor, *n* (%)	5 (2)	326 (49)	<0.001
Diuretic, *n* (%)	9 (3)	261 (40)	<0.001

^1^ Data are presented as mean ± SD, median (IQR) and absolute number (percentage) for normally distributed, skewed and nominal data, respectively. ^2^ *p*-value for difference was tested by *t* and Mann-Whitney tests for normally and skewed distributed continuous variables, respectively, and Chi-Square tests for nominal variables. ^3^ Intake of niacin equivalents was calculated by adding up niacin and one-sixtieth of tryptophan intake. Subjects who were using niacin supplementation were excluded. eGFR, estimated glomerular filtration rate; HbA1c, hemoglobin A1c; *N*^1^-MN, *N*^1^-methylnicotinamide; RTR, renal transplant recipients.

**Table 2:** Association of RTR and healthy kidney donors grouping with *N*^1^-MN excretion ^1^.

Variable	Model 1 ^2^		Model 2 ^3^		Model 3 ^4^		Model 4 ^5^	
	Std.β	*p*-Value	Std.β	*p*-Value	Std.β	*p*-Value	Std.β	*p*-Value
Grouping	−0.42	<0.001	−0.44	<0.001	−0.25	<0.001	−0.21	<0.001
Sex	-	-	−0.15	<0.001	−0.14	<0.001	−0.10	0.002
Age, years	-	-	−0.16	<0.001	−0.11	<0.001	−0.07	0.02
eGFR, ml/min/1.73 m^2^	-	-	-	-	0.31	<0.001	0.29	<0.001
Energy intake, kcal/day	-	-	-	-	-	-	−0.10	0.08
Tryptophan intake, mg/day	-	-	-	-	-	-	0.007	0.91
Niacin intake, mg/day	-	-	-	-	-	-	0.25	<0.001
Plasma vitamin B_6_, nmol/L	-	-	-	-	-	-	0.23	<0.001
R^2^	0.18		0.23		0.28		0.37	

^1^ Linear regression analyses were performed to investigate the association of RTR and healthy kidney donors as grouping variable with *N*^1^-MN excretion, with adjustment for potential confounders. ^2^ Model 1: crude model. ^3^ Model 2: adjusted for age and sex. ^4^ Model 3: adjusted as for model 2 and for eGFR. ^5^ Model 4: adjusted as for model 3 and for intake of energy, tryptophan and niacin and plasma vitamin B_6_. eGFR, estimated glomerular filtration rate; *N*^1^-MN, *N*^1^-methylnicotinamide; RTR, renal transplant recipients; std.β, standardized beta coefficient.

**Table 3:** Baseline characteristics of RTR across tertiles of *N*^1^-MN excretion stratified by sex ^1^.

Variable	Tertiles of Sex-Stratified *N*^1^-MN Excretion			*p*-Value ^2^
	T1 *n* = 219	T2 *n* = 221	T3 *n* = 220	
Males, μmol/day	<19.2	19.2–28.8	>28.8	
Females, μmol/day	<16.1	16.1–25.6	>25.6	
Male, *n* (%)	126 (58)	127 (58)	126 (57)	-
Age, years	54.6 ± 12.7	53.7 ± 13.1	50.7 ± 12.1	0.004
BMI, kg/m^2^	25.8 (22.7–29.4)	26.1 (23.3–29.0)	26.0 (23.6–29.6)	0.41
Body surface area, m^2^	1.9 ± 0.2	1.9 ± 0.2	2.0 ± 0.2	0.13
Lifestyle				
Current smoker, *n* (%)	21 (10)	25 (11)	32 (15)	0.26
Alcohol consumption, g/day	0.5 (0.0–7.0)	3.2 (0.1–11.3)	6.7 (0.8–20.9)	<0.001
Vegetarian, *n* (%)	7 (3)	2 (1)	3 (1)	0.16
Dietary intake				
Energy, kcal/day	2065 ± 586	2197 ± 675	2285 ± 647	0.002
Tryptophan, mg/day	1001 ± 253	1063 ± 273	1112 ± 274	<0.001
Niacin, mg/day	16.6 ± 4.9	17.6 ± 4.8	19.5 ± 5.5	<0.001
Plasma vitamin B_6_, nmol/L	20.3 (14.0–39.0)	29.5 (19.0–47.0)	39.0 (22.0–65.0)	<0.001
Hemodynamic				
Systolic blood pressure, mmHg	139 ± 18	134 ± 18	135 ± 16	0.01
Diastolic blood pressure, mmHg	83 ± 11	82 ± 12	83 ± 11	0.20
Mean arterial pressure, mmHg	109 ± 15	106 ± 15	106 ± 14	0.07
Heart rate, beats per minute	69 ± 11	68 ± 12	68 ± 12	0.52
Antihypertensive use, *n* (%)	199 (91)	193 (87)	189 (86)	0.26
Lipids				
Total cholesterol, mmol/L	5.1 ± 1.2	5.2 ± 1.1	5.0 ± 1.1	0.36
HDL, mmol/L	1.3 (1.0–1.6)	1.3 (1.1–1.6)	1.3 (1.1–1.7)	0.06
LDL, mmol/L	3.0 ± 0.9	3.1 ± 0.9	2.9 ± 0.9	0.31
Triglycerides, mmol/L	1.7 (1.3–2.3)	1.7 (1.3–2.3)	1.6 (1.1–2.2)	0.03
Statin, *n* (%)	122 (56)	115 (52)	112 (51)	0.55
Glucose homeostasis				
Glucose, mmol/L	5.3 (4.8–6.0)	5.3 (4.8–5.9)	5.2 (4.7–6.2)	0.58
HbA1c, (%)	5.8 (5.5–6.3)	5.9 (5.6–6.1)	5.7 (5.4–6.1)	0.05
Diabetes, *n* (%)	58 (27)	44 (20)	50 (23)	0.26
Antidiabetic, *n* (%)	41 (19)	28 (13)	27 (12)	0.10
Other serum parameters				
Hs-CRP, mg/L	1.7 (0.8–5.3)	1.6 (0.6–3.8)	1.4 (0.7–4.6)	0.42
Phosphate, mmol/L	1.0 ± 0.2	1.0 ± 0.2	0.9 ± 0.2	0.01
Immunosuppressant medication				
Prednisolon dose, mg/day	10 (7.5–10)	10 (7.5–10)	10 (7.5–10)	0.18
Calcineurin inhibitor, *n* (%)	136 (62)	125 (57)	112 (51)	0.06
Cyclosporine, *n* (%)	87 (40)	82 (37)	84 (38)	0.85
Azathioprine, *n* (%)	35 (16)	36 (16)	41 (19)	0.72
Proliferation inhibitor, *n* (%)	171 (78)	186 (84)	191 (87)	0.04
Other medication				
Acetylsalicylic acid, *n* (%)	55 (25)	47 (21)	25 (11)	0.001
Anticonvulsant, *n* (%)	7 (3)	5 (2)	7 (3)	0.80
Proton pump inhibitor, *n* (%)	127 (58)	107 (48)	92 (42)	0.003
Diuretic, *n* (%)	104 (48)	79 (36)	78 (36)	0.01
Kidney function				
Serum creatinine, μmol/L	138 (104–189)	122 (101–153)	114 (94–140)	<0.001
Cystatin C, mg/L	2.0 (1.4–2.8)	1.6 (1.3–2.1)	1.4 (1.2–1.9)	<0.001
eGFR, ml/min/1.73 m^2^	39.0 ± 18.7	45.8 ± 16.9	52.7 ± 18.0	<0.001
Proteinuria ≥ 0.5 g/day, *n* (%)	55 (25)	39 (18)	38 (17)	0.07
Kidney transplantation				
Time since transplantation, years	5.6 (1.7–12.9)	5.0 (1.5–11.0)	6.5 (2.9–12.3)	0.16
Donor				
Age, years	46 (33–54)	47 (29–57)	43 (29–53)	0.22
Male, *n* (%)	104 (48)	110 (50)	112 (51)	0.60
Post mortem status, *n* (%)	161 (74)	143 (65)	121 (55)	<0.001
Primary kidney disease				
Primary glomerular disease, *n* (%)	48 (22)	67 (30)	71 (32)	0.04
Glomerulonephritis, *n* (%)	12 (6)	17 (8)	21 (10)	0.27
Tubulointerstitial disease, *n* (%)	27 (12)	30 (14)	20 (9)	0.32
Polycystic renal disease, *n* (%)	52 (24)	45 (20)	40 (18)	0.35
Dysplasia and hypoplasia, *n* (%)	9 (4)	10 (5)	9 (4)	0.97
Renovascular disease, *n* (%)	17 (8)	8 (4)	11 (5)	0.15
Diabetic nephropathy, *n* (%)	15 (7)	7 (3)	13 (6)	0.20
Other or unknown cause, *n* (%)	39 (18)	36 (16)	35 (16)	0.85

^1^ Data are presented as mean ± SD, median (IQR) and absolute number (percentage) for normally distributed, skewed and nominal data, respectively. ^2^ *p*-value for difference was tested by ANOVA and Kruskal-Wallis tests for normally and skewed distributed continuous variables, respectively, and Chi-Square tests for nominal variables. eGFR, estimated glomerular filtration rate; HbA1c, hemoglobin A1c; hs-CRP, high-sensitivity C-reactive protein; *N*^1^-MN, *N*^1^-methylnicotinamide; RTR, renal transplant recipients.

**Table 4:** Association of *N*^1^-MN excretion with risk of all-cause mortality in RTR ^1^.

Model	*N*^1^-MN Excretion (log_2_) As Continuous Variable *n* = 660		Tertiles of Sex-Stratified *N*^1^-MN Excretion ^2^				
			T1 *n* = 219		T2 *n* = 221		T3 *n* = 220
	HR (95% CI)	*p*-Value	HR (95% CI)	*p*-Value	HR (95% CI)	*p*-Value	Reference HR
1 ^3^	0.53 (0.43–0.65)	<0.001	3.28 (2.04–5.26)	<0.001	2.41 (1.48–3.93)	<0.001	1.00
2 ^4^	0.57 (0.45–0.71)	<0.001	2.68 (1.67–4.33)	<0.001	2.04 (1.25–3.34)	0.004	1.00
3 ^5^	0.59 (0.47–0.74)	<0.001	2.65 (1.60–4.39)	<0.001	2.10 (1.25–3.52)	0.005	1.00
4 ^6^	0.69 (0.53–0.90)	0.005	2.10 (1.17–3.78)	0.01	2.04 (1.15–3.63	0.02	1.00
5 ^7^	0.75 (0.58–0.96)	0.02	1.86 (1.07–3.25)	0.02	1.80 (1.04–3.13)	0.04	1.00
6 ^8^	0.65 (0.51–0.82)	<0.001	2.25 (1.35–3.75)	0.002	2.06 (1.23–3.46)	0.006	1.00
7 ^9^	0.60 (0.48–0.76)	<0.001	2.59 (1.54–4.35)	<0.001	2.13 (1.26–3.61)	0.005	1.00
Events (*n*)	143		67		53		23

^1^ Cox regression analyses were performed to investigate the association of *N*^1^-MN excretion with risk of all-cause mortality in RTR, with adjustment for potential confounders. ^2^ *N*^1^-MN excretion was <19.2, 19. 2–28.8, and >28.8 μmol/day for males, and <16.1, 16.1–25.6, and >25.6 μmol/day for females in T1, T2, and T3, respectively. ^3^ Model 1: not adjusted in tertiles of sex-stratified *N*^1^-MN excretion, adjusted for sex in continuous analyses. ^4^ Model 2: adjusted as for model 1 and for age. ^5^ Model 3: adjusted as for model 2 and for smoking and body surface area. ^6^ Model 4: adjusted as for model 3 and for intake of alcohol and energy and plasma vitamin B_6_. ^7^ Model 5: adjusted as for model 3 and for eGFR, proteinuria, donor status and primary glomerular disease. ^8^ Model 6: adjusted as for model 3 and for use of proliferation inhibitors, acetylsalicylic acid, proton pump inhibitors and diuretics. ^9^ Model 7: adjusted as for model 3 and for hs-CRP. eGFR, estimated glomerular filtration rate; hs-CRP, high-sensitivity C-reactive protein; *N*^1^-MN, *N*^1^-methylnicotinamide; RTR, renal transplant recipients.

## Supplementary Materials

The following is available online at https://www.mdpi.com/2077-0383/8/11/1948/s1, Figure S1: Flow of participants through study protocol.
