# Supplementary material for: Urinary Excretion of N1-Methylnicotinamide, as a Biomarker of Niacin Status, and Mortality in Renal Transplant Recipients
Source: J Clin Med. 2019 Nov 12;8(11):1948. doi: 10.3390/jcm8111948 (PMC6912198; doi:10.3390/jcm8111948)

Supplementary Materials

Supplementary Figure S1: Flow of participants through study protocol

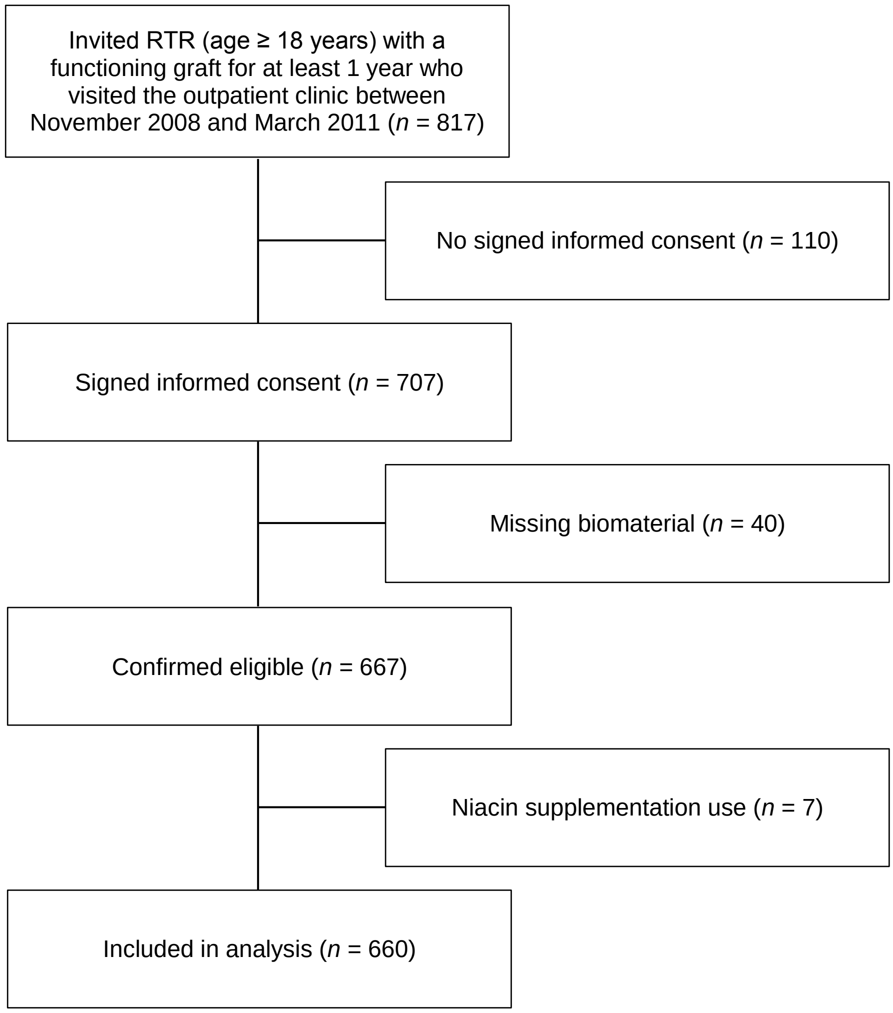

Supplement: Supplementary file 1 [file jcm-08-01948-s001.pdf]
